# Alendronate treatment for osteogenesis imperfecta type III in twin babies: a case report

**DOI:** 10.1186/1687-9856-2013-S1-P158

**Published:** 2013-10-03

**Authors:** Annang Giri Moelyo, Septin Widiretnani, Ani Suryandari

**Affiliations:** 1 Child Health Department, Faculty of Medicine Sebelas Maret University Moewardi Hospital, Surakarta, Indonesia

## 

Osteogenesis Imperfecta (OI) is a connective tissue disorders. There are several types of OI. OI can be autosomal dominant, some cases can be found in recessive by mosaicism in the parent. The main goal of treatment is to reduce the incidence of fractures, prevent deformities of bones and scoliosis, and improve the functional patient. This is a case of OI given alendronate as an alternative treatment.

Twin boys babies born in Moewardi Hospital by sectio caesaria. Baby I and II seemed swollen on both of hand and leg. Both of the babies appeared to be less active and limitation of range of motion, with blue sclera. Result of x-rays, it appeared multiple fractures in extremities and costae. We diagnosed osteogenesis omperfecta type III, clinically. We gave alendronate 1 x 1 mg orally, because sodium pamidronate was not available at that time. Alendronate were given by thirty to forty five degree position. Orthopedic surgeon gave on conservative therapy by slap. During treatment in hospital, appeared new fractures in each baby. After giving alendronate therapy for 4 months, the baby I didn’t show new fractures, the baby II suffered three new fractures. There were no side effect of alendronate treatment on gastrointestinal. At the aged of 5th month, the patients were given intravenously sodium pamidronate for 3 days with a dose of 2 mg. Three months after pamidronate, the baby I didn't show new fractures, the baby II suffered one new fracture. Bone mineral density was not performed in these patients. We conclude that oral biphosphonate could be alternative treatment for OI if intravenous biphosphonate is unavailable. It still needs more reseach regarding to effectiveness of oral biphosphonate compare to intravenous biphosphonate.

